# Incorporating Behavioral Science in Medication Adherence Communication: A Randomized Clinical Trial

**DOI:** 10.1001/jamanetworkopen.2025.10162

**Published:** 2025-05-14

**Authors:** Punam Keller, Ted Robertson, Lee-Sien Kao, Yong Li, Brandon Merrell, Linda Chung, Emily Boudreau, Alex James, Lauren Esterly, Kaitlin Hanken, Niteesh K. Choudhry

**Affiliations:** 1 Tuck School of Business, Dartmouth College, Hanover, New Hampshire; 2 ideas42, Washington, DC; 3 Humana Inc, Louisville, Kentucky; 4 Center for Healthcare Delivery Sciences, Division of Pharmacoepidemiology and Pharmacoeconomics, Department of Medicine, Brigham and Women’s Hospital, Boston, Massachusetts; 5 Center for Healthcare Delivery Sciences, Department of Medicine, Brigham and Women’s Hospital, Harvard Medical School, Boston, Massachusetts

## Abstract

**Importance:**

Hypertension increases the risk of myocardial infarction and strokes, but adherence to antihypertensive medications remains suboptimal. Increasing medication adherence may help prevent hypertension-related disability and death.

**Objective:**

To estimate the effect of incorporating behavioral science principles in mailed communications intended to increase hypertension medication adherence.

**Design, Setting, and Participants:**

This randomized clinical trial was conducted from August 18 to December 31, 2023, at Humana, a large health insurance provider, and included Medicare Advantage beneficiaries aged 65 to 80 years who were taking hypertension medications with adherence rates of 60% to 85%. Data were extracted for the as-treated analysis on April 14, 2024.

**Intervention:**

Study participants were randomized in equal proportions to 1 of 7 groups: 6 received mailed communications that incorporated combinations of behavioral science principles, and 1 received no mailing (control, or usual care condition). The specific principles included were social norming (the mailing noted that more Humana beneficiaries each year were improving their medication adherence scores), messenger effects (the mailing signatory was a pharmacist), and processing fluency (the mailing incorporated a visual circle metaphor indicating current and complete adherence to prompt “closing the ring”). The first mailing contained the participant’s baseline refill score, and a second mailing noted any changes in the participant’s refill score after 60 days.

**Main Outcomes and Measures:**

The primary outcome was end-of-year hypertension medication adherence calculated from pharmacy claims data using the proportion of days covered (PDC) metric (having medication available for 1 target hypertension drug, as measured through prescription claims, counted as adherent on a given day). A secondary outcome was the proportion of study participants with end-of-year PDC of 80% or greater.

**Results:**

The study participants (n = 64 290) had a mean (SD) age of 71.4 (4.1) years, and 54.1% were female. Mean (SD) end-of-year adherence in the control group was 0.81 (0.12) and was no different in any of the intervention arms (absolute mean difference compared with control was 0.00 [95% CI, −0.01 to 0.01]). The proportion of participants with an end-of-year PDC greater than 80% also did not differ significantly between the control group (6587 of 9183 [71.7%]) and any of the intervention arms (range, 6467 of 9180 [70.4%] to 6598 of 9177 [71.9%]).

**Conclusions and Relevance:**

In this randomized clinical trial, use of social norms, messenger effects, and processing fluency in mailed communications did not significantly increase hypertension medication adherence. Further research is needed to identify tactics to effectively increase adherence.

**Trial Registration:**

ClinicalTrials.gov Identifier: NCT06066541

## Key Points

**Question:**

Can combinations of behavioral science principles in messaging increase hypertension medication adherence?

**Findings:**

In this randomized clinical trial that included 64 290 adults aged 65 to 80 years, there were no statistically significant differences in hypertension medication adherence between those who received mailed communications that incorporated social norms, messenger effects, and processing fluency and those who received no mailing.

**Meaning:**

Further research is needed to identify tactics to effectively increase hypertension medication adherence.

## Teaser

This randomized clinical trial investigates whether including behavioral science principles in mailed messaging increases hypertension medication adherence among adults aged 65 to 80 years.

## Introduction

Hypertension substantially increases the risk of myocardial infarction and stroke,^1^ but medications that can effectively control hypertension are not always taken as prescribed; adherence rates for hypertension medications typically range from 40% to 74%.^2^ Nonadherence is a potent contributor to preventable disability and death among middle-aged and older adults.^3^ The asymptomatic nature of hypertension makes medication adherence especially challenging, as there are no physical symptoms that could serve as a natural cue to take any prescribed hypertension medications. Therefore, innovative strategies for improving hypertension medication adherence are particularly needed. Many strategies have been tested to increase medication adherence, including patient education, medication regimen management, pharmacist comanagement, cognitive behavioral therapy, reminders, and incentives.^4^ However, effects on adherence are generally modest.^5,6^

Communication strategies using behavioral science theories may help to improve medication adherence^7^ but have not been well studied for hypertension medications, especially in mailed communications. We hypothesized that 3 specific behavioral science principles may be particularly impactful in this context. First, making social norms or information about others’ behavior more salient can encourage an individual’s own behaviors when base rates of those behaviors are high. When base rates are low, dynamic norms, or information about how others’ behavior is changing (in particular, that medication adherence is improving), may be more impactful.^8^ Dynamic norms have shown impact for a range of healthy behaviors.^9^ Second, the person or entity who is communicating a message may influence its effectiveness based on factors such as how trusted and credible the messenger is or how much the messenger has in common with the people with whom they are communicating.^10^ Including a specific prescription-related message from a pharmacist, a trusted medication expert, may increase the impact of the mailing, especially if the pharmacist is taking the same medications. Third, processing fluency—the subjective ease with which information is understood—can influence its actionability. Increasing processing fluency has been impactful for exercise^11^ and other health behaviors,^12^ including treatment adherence for juvenile idiopathic arthritis^13^ and cancer screening.^14^ Studies providing patients with summary information for statin medication adherence have demonstrated null^15^ or limited^16^ results, but incorporating measures to ease processing fluency through tactics such as visual metaphors that emphasize goal progress and attainability may increase the impact, especially when low health literacy is a key barrier to medication adherence.^17^ In this study, we tested the application of these theories to hypertension medication adherence for a Medicare population aged 65 to 80 years.

## Methods

### Study Design

We conducted a 7-arm, open-label randomized clinical trial. The Advarra institutional review board approved this trial, providing a waiver of informed consent because of the minimal-risk nature of the study. The study is registered (NCT06066541) and was overseen by a National Institutes of Health–appointed data and safety monitoring board. We followed the Consolidated Standards of Reporting Trials (CONSORT) reporting guideline.

The trial protocol is available in Supplement 1. The study was initiated with the first participant outreach on August 18, 2023, with follow-up ending December 31, 2023, and data extraction for analysis on April 14, 2024.

### Study Setting and Population

The study was conducted at Humana, a large national health insurer. Participants were Medicare Advantage beneficiaries 65 to 80 years of age who (1) had filled 2 or more prescriptions for angiotensin-converting enzyme inhibitors or angiotensin receptor blockers in 2023 prior to the study start, (2) had an adherence level between 60% and 85% as measured by proportion of days covered (PDC) in prescription insurance claims following the Medicare Star Ratings approach,^18^ and (3) had provided addresses to Humana that could be used for mailed communications. The exclusion of adults older than 80 years was scientifically justified because patients in that age group are at higher risk for comorbid events, including mortality, and clinical hypertension guidelines recommend different considerations for hypertension medications for patients older than 80 years due to differences in likelihood and type of adverse effects and interactions with other antihypertensive drugs. Therefore, the increased risk among these patients has different implications for hypertension medication adherence and would warrant a separate study for patients aged 80 years or older. Per Humana policy, beneficiaries of specific insurance products (eg, employer group Medicare for retirees) were also excluded from the study. Study participants were only included in the analysis if they remained enrolled at the time of data extraction (April 14, 2024).

PDC is an objective measure of medication adherence calculated with prescription claims data. The current study included participants eligible for the Medicare Star Ratings hypertension medication adherence measure,^18^ which targets angiotensin-converting enzyme inhibitors or angiotensin receptor blockers. Within these therapeutic classes, only 1 medication is usually prescribed at a time, as taking multiple medications within these 2 classes is a safety concern. A participant having medication available (measured through prescription claims) for 1 target hypertension drug was counted as covered (adherent) on a given day. Unlike similar calculations, such as the medication possession ratio, PDC adjusts for early prescription fills to avoid overreporting of adherence.

### Interventions and Randomization

The primary component of our intervention was a mailed individualized communication sent from Humana that reported the study participant’s medication adherence as a personalized refill score, which was calculated using the PDC metric. We stratified potentially eligible participants prior to randomization based on the length of their refill cycle (30 or 90 days) and whether they had drugs available at the time of randomization. Within each of the 4 resulting strata, participants were randomized to 1 of 6 intervention arms or the control arm in an equal (1:1:1:1:1:1:1) ratio. The randomization process was conducted using proc surveyselect with a fixed seed in SAS, version 8.3 (SAS Institute Inc). Participants randomized to each of the intervention arms received their version of the adherence scorecard according to protocol. Randomization was conducted August 18, 2023.

Participants in arm 1 were sent the baseline mailing, which included the participant’s medication refill score displayed on a vertical color-coded bar, with information about the meaning of the score, how it was calculated, and the benefits of taking medications as prescribed. It also contained tips to improve their refill score. Participants in arm 2 were sent a modified version of the baseline mailing that included a dynamic social norm indicating that “more people each year are improving their refill scores by refilling on time and taking their meds as prescribed, which may help them better manage their high blood pressure.” Participants in arm 3 were sent a modified version of the baseline mailing leveraging messenger effects by including an additional testimonial from and signed by a Humana pharmacist taking the same medication about their own challenges with taking medications on time and the importance of doing so. Participants in arm 4 were sent a modified version of the baseline mailing that increased processing fluency by using an alternative visual circle metaphor indicating current and complete adherence to prompt “closing the ring.” Participants in arm 5 were sent a letter with the modified version of the processing fluency mailing that also included the dynamic social norms. Participants in arm 6 were sent the modified version of the processing fluency mailing that also included the messenger effects. Participants in arm 7 did not receive a mailing (usual care) (Figure).

**Figure.**
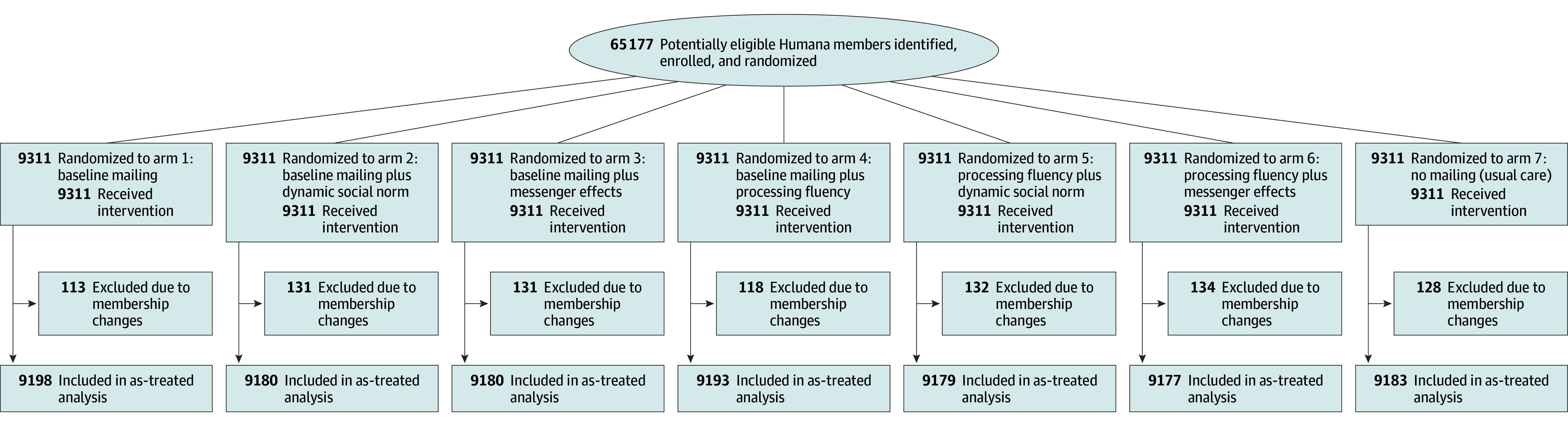
CONSORT Diagram

Two mailings were sent during the intervention, with participants remaining in the arm to which they were originally randomized. The first mailing contained their refill score at the time of randomization. The second mailing was sent within 60 days of the first message and noted any changes in the participant’s refill score.

### Outcomes

The primary prespecified outcome measure was medication adherence, assessed with PDC based on prescription claims data at the end of the calendar year. This period was chosen to replicate how the Medicare program assesses performance on Star Ratings. The secondary outcome was the proportion of participants who were fully adherent at the end of 2023 (measured for the full calendar year), defined as a PDC of at least 80% based on the threshold used by the Centers for Medicare & Medicaid Services.^18^

Post hoc outcomes included shorter-term outcome measures. The shorter-term outcome measures included postintervention PDC of at least 80%, measured between the time of the intervention and December 2023; refill by the last date that the individual had medication available; number of days to medication refill after the first mailing was sent; and number of days to medication refill after the second mailing was sent.

### Statistical Analysis

We estimated that a sample size of 59 997 (8571 per arm) would provide 80% power to observe a difference of approximately 1 percentage point in end-of-year PDC (which would be a clinically significant change) compared with the usual care arm, conservatively assuming a usual care arm rate of 70% and a Holm-Bonferroni corrected α of .05 to adjust the familywise error rates for multiple hypotheses testing. We randomized participants before our study launched, with recruitment starting in August 2023. We recruited in 2 waves (on August 18, 2023, and August 24, 2023) to ensure a sufficient sample size after adjusting for dropouts due to loss of eligibility or changes in Humana Medicare Advantage membership.

We reported the means and frequencies of prerandomization variables separately by arm, and we tested the overall difference in these variables between arms. Race and ethnicity, ascertained by patient self-report from Medicare enrollment files, were included as a variable in the analysis because of their previously reported association with medication adherence. Categories were Black, White, underrepresented (included American Indian or Alaska Native, Asian, Hispanic, Pacific Islander, or other race and ethnicity), and unknown.

To quantify the effects of the intervention, we used linear regression for continuous outcomes (end-of-year medication adherence, number of days to refill) and logistic regression for binary outcomes (end-of-year adherence rate, rate of refill by the last date of medication available). All analyses compared each arm independently with the control group while controlling for design factors (refill cycle and drugs available).

Subgroup analyses were performed by age (65-69, 70-74, and 75-80 years), sex, eligibility for low-income subsidies or dual eligibility, baseline adherence (PDC<78% vs ≥78% as the median adherence, rather than the standard 80% cutoff), and baseline refill cycle (30 days vs 90 days). As-treated analyses were conducted using SAS Enterprise Guide, version 8.3 (SAS Institute Inc). Two-sided *P* < .05 was considered significant.

## Results

We randomized 65 177 Medicare Advantage beneficiaries before our study launched. After excluding 887 for membership changes, the final sample consisted of 64 290 participants (Figure and eFigure in Supplement 2). These participants had an overall mean (SD) age of 71.4 (4.1) years; 54.1% were female, and 45.9% were male. A total of 23.7% were Black, 64.0% were White, 8.9% were of underrepresented race and ethnicity, and 3.4% had unknown race and ethnicity. In all, 78.6% had a 90-day refill cycle. Other baseline characteristics by study arm are shown in Table 1. The cohort was well balanced across study arms.

**Table 1.** Characteristics of the Study Population

Characteristic	Participants^a^						
	Arm 1: baseline mailing (n = 9198)	Arm 2: baseline plus dynamic social norm (n = 9180)	Arm 3: baseline plus messenger effects (n = 9180)	Arm 4: processing fluency (n = 9193)	Arm 5: processing fluency plus dynamic social norm (n = 9179)	Arm 6: processing fluency plus messenger effects (n = 9177)	Arm 7: control (no mailing) (n = 9183)
Sex							
Female	4881 (53.1)	5005 (54.5)	5041 (54.9)	4938 (53.7)	5040 (54.9)	4967 (54.1)	4939 (53.8)
Male	4317 (46.9)	4175 (45.5)	4139 (45.1)	4255 (46.3)	4139 (45.1)	4210 (45.9)	4244 (46.2)
Race and ethnicity							
Black	2229 (24.2)	2140 (23.3)	2225 (24.2)	2218 (24.1)	2114 (23.0)	2143 (23.4)	2168 (23.6)
White	5882 (63.9)	5914 (64.4)	5822 (63.4)	5837 (63.5)	5903 (64.3)	5900 (64.3)	5879 (64.0)
Underrepresented^b^	778 (8.5)	820 (8.9)	808 (8.8)	814 (8.9)	842 (9.2)	845 (9.2)	829 (9.0)
Unknown	309 (3.4)	306 (3.3)	325 (3.5)	324 (3.5)	320 (3.5)	289 (3.1)	307 (3.3)
Refill cycle, d							
30	1965 (21.4)	1962 (21.4)	1961 (21.4)	1977 (21.5)	1974 (21.5)	1972 (21.5)	1975 (21.5)
90	7223 (78.5)	7218 (78.6)	7219 (78.6)	7216 (78.5)	7205 (78.5)	7205 (78.5)	7208 (78.5)
Age, mean (SD), y	71.36 (4.11)	71.44 (4.09)	71.44 (4.09)	71.33 (4.07)	71.49 (4.10)	71.44 (4.07)	71.50 (4.07)
Geographic region							
Northeast	503 (5.5)	509 (5.5)	490 (5.3)	496 (5.4)	465 (5.1)	532 (5.8)	524 (5.7)
Midwest	1629 (17.7)	1690 (18.4)	1627 (17.7)	1735 (18.9)	1691 (18.4)	1606 (17.5)	1621 (17.7)
South	5902 (64.2)	5795 (63.1)	5894 (64.2)	5763 (62.7)	5832 (63.6)	5928 (64.6)	5840 (63.6)
West	1162 (12.6)	1186 (12.9)	1167 (12.7)	1198 (13.0)	1188 (12.9)	1111 (12.1)	1196 (13.0)
Population density							
Urban	5450 (59.3)	5463 (59.5)	5343 (58.2)	5445 (59.2)	5446 (59.3)	5385 (58.7)	5376 (58.5)
Suburban	2396 (26.0)	2298 (25.0)	2391 (26.0)	2384 (25.9)	2364 (25.8)	2375 (25.9)	2409 (26.2)
Rural	1167 (12.7)	1230 (13.4)	1276 (13.9)	1200 (13.1)	1203 (13.1)	1231 (13.4)	1206 (13.1)
Unknown	185 (2.0)	189 (2.1)	170 (1.9)	164 (1.8)	166 (1.8)	186 (2.0)	192 (2.1)
LIS or dual eligibility status							
LIS only	497 (5.4)	518 (5.6)	536 (5.8)	514 (5.6)	524 (5.7)	567 (6.2)	566 (6.2)
Medicaid eligible only	9 (0.1)	7 (0.1)	10 (0.1)	12 (0.1)	16 (0.2)	8 (0.1)	10 (0.1)
LIS and dual eligibility	2407 (26.2)	2364 (25.8)	2289 (24.9)	2396 (26.1)	2330 (25.4)	2294 (25.0)	2296 (25.0)
No LIS or dual eligibility	6285 (68.3)	6291 (68.5)	6345 (69.1)	6271 (68.2)	6309 (68.7)	6308 (68.7)	6311 (68.7)
Hypertension PDC as of selection, mean (SD)	0.77 (0.06)	0.77 (0.06)	0.77 (0.06)	0.77 (0.06)	0.77 (0.06)	0.77 (0.06)	0.77 (0.06)
Hypertension PDC ≥80% at selection	3690 (40.1)	3615 (39.4)	3705 (40.4)	3720 (40.5)	3690 (40.2)	3650 (39.8)	3722 (40.5)
Unique prescription fills for hypertension medication at selection, mean (SD), No.	2.59 (1.26)	2.59 (1.25)	2.59 (1.22)	2.60 (1.25)	2.59 (1.19)	2.60 (1.21)	2.59 (1.20)
Elixhauser Comorbidity Index, mean (SD)^c^	3.34 (2.79)	3.33 (2.73)	3.29 (2.74)	3.35 (2.79)	3.25 (2.73)	3.33 (2.74)	3.31 (2.76)
Resource utilization, mean (SD), No.							
Days hospitalized	1.54 (7.30)	1.34 (6.03)	1.38 (5.60)	1.54 (6.29)	1.41 (6.38)	1.45 (6.46)	1.45 (5.84)
ED visits	0.57 (1.36)	0.55 (1.35)	0.53 (1.24)	0.57 (1.37)	0.55 (1.56)	0.57 (1.76)	0.53 (1.27)
Office visits	10.40 (10.26)	10.55 (10.99)	10.41 (10.50)	10.46 (10.28)	10.41 (10.65)	10.47 (10.64)	10.44 (10.33)
Unique medications filled	11.36 (6.44)	11.39 (6.40)	11.39 (6.43)	11.42 (6.34)	11.24 (6.39)	11.41 (6.39)	11.50 (6.47)

Abbreviations: ED, emergency department; LIS, low income subsidy; PDC, proportion of days covered.

^a^ Data are presented as number (percentage) of participants unless otherwise indicated.

^b^ Includes American Indian or Alaska Native, Asian, Hispanic, Pacific Islander, or other race and ethnicity.

^c^ Ranges from 0 to 31, with higher scores indicating a greater number of clinical conditions.

As shown in Table 2, study participants across intervention arms did not demonstrate statistically significant differences in our primary or secondary outcomes of interest. Mean (SD) end-of-year PDC in treatment arms 1 through 6 was 0.81 (0.12) (absolute difference, 0.00; 95% CI, −0.01 to 0.01) in each arm compared with a mean (SD) of 0.81 (0.12) in the reference group (arm 7). End-of-year adherence, defined as PDC of 80% or greater at the end of 2023, also did not vary significantly across arms (rates ranged from 6467 of 9180 [70.4%] in arm 3 to 6598 of 9177 [71.9%] in arm 6) compared with a base rate of 6587 of 9183 (71.7%) in arm 7 (Table 2).

**Table 2.** Changes in Medication Adherence Associated With Intervention

Outcome	Arm 1: baseline mailing		Arm 2: baseline plus dynamic social norm		Arm 3: baseline plus messenger effects		Arm 4: processing fluency		Arm 5: processing fluency plus dynamic social norm		Arm 6: processing fluency plus messenger effects		Arm 7: control (no mailing)	
	Mean (SD)^a^	Absolute difference (95% CI)	Mean (SD)^a^	Absolute difference (95% CI)	Mean (SD)^a^	Absolute difference (95% CI)	Mean (SD)^a^	Absolute difference (95% CI)	Mean (SD)^a^	Absolute difference (95% CI)	Mean (SD)^a^	Absolute difference (95% CI)	Mean (SD)^a^	Absolute difference (95% CI)
**Primary outcome**														
Hypertension PDC at end of 2023	0.81 (0.12)	0.00 (−0.01 to 0.00)	0.81 (0.12)	0.00 (−0.01 to 0.01)	0.81 (0.12)	0.00 (−0.01 to 0.00)	0.81 (0.12)	0.00 (−0.01 to 0.00)	0.81 (0.12)	0.00 (0.00 to 0.01)	0.81 (0.12)	0.00 (−0.01 to 0.01)	0.81 (0.12)	[Reference]
**Secondary outcome**														
Participants with hypertension PDC≥80% at end of 2023, No. (%)	6518 (70.9)	0.95 (0.86 to 1.06)	6590 (71.8)	1.00 (0.90 to 1.11)	6467 (70.4)	0.93 (0.84 to 1.03)	6515 (70.9)	0.96 (0.86 to 1.06)	6558 (71.4)	0.99 (0.89 to 1.09)	6598 (71.9)	1.01 (0.91 to 1.12)	6587 (71.7)	[Reference]
**Post hoc outcomes**														
Days to refill, first scorecard	47.9 (29.7)	0.05 (−1.37 to 1.47)	47.8 (29.8)	−0.12 (−1.53 to 1.30)	48.2 (30.1)	0.32 (−1.10 to 1.74)	48.2 (30.6)	0.31 (−1.11 to 1.73)	48.6 (30.6)	0.76 (−0.66 to 2.17)	47.6 (30.0)	−0.33 (−1.75 to 1.08)	47.9 (30.0)	[Reference]
Days to refill, second scorecard	33.1 (18.8)	0.03 (−1.05 to 1.11)	33.0 (18.7)	−0.07 (−1.14 to 1.01)	33.0 (18.6)	−0.05 (−1.13 to 1.03)	32.6 (18.8)	−0.40 (−1.47 to 0.68)	33.2 (18.6)	0.11 (−0.96 to 1.19)	33.4 (18.9)	0.36 (−0.72 to 1.44)	33.0 (18.8)	[Reference]

Abbreviation: PDC, proportion of days covered.

^a^ Data are presented as the mean (SD) value unless otherwise indicated.

Post hoc analyses yielded similar results. The mean (SD) number of days to refill after the first scorecard (defined as the enrollment date) was 47.9 (29.7) in the reference group (arm 7), with effect sizes of 0.05 (95% CI, −1.37 to 1.47) days in arm 1, −0.12 (95% CI, −1.53 to 1.30) days in arm 2, 0.32 (95% CI, −1.10 to 1.74) days in arm 3, 0.31 (95% CI, −1.11 to 1.73) days in arm 4, 0.76 (95% CI, −0.66 to 2.17) days in arm 5, and −0.33 (95% CI, −1.75 to 1.08) days in arm 6. The mean (SD) number of days to refill after the second scorecard (defined as the date of the second scorecard: October 26, 2023) was 33.0 (18.88) for study participants in the reference group (arm 7). In comparison, the mean differed by 0.03 (95% CI, −1.05 to 1.11) days in arm 1, −0.07 (95% CI, −1.14 to 1.01) days in arm 2, −0.05 (95% CI, −1.13 to 1.03) days in arm 3, −0.40 (95% CI, −1.47 to 0.68) days in arm 4, 0.11 (95% CI, −0.96 to 1.19) days in arm 5, and 0.36 (95% CI, −0.72 to 1.44) days in arm 6. The odds ratios for a medication refill by the last day of available medication (established at the time of enrollment) were 0.98 (95% CI, 0.90-1.08) in arm 1, 1.06 (95% CI, 0.96-1.16) in arm 2, 1.02 (95% CI, 0.93-1.12) in arm 3, 1.02 (95% CI, 0.93-1.12) in arm 4, 0.99 (95% CI, 0.90-1.09) in arm 5, and 1.03 (95% CI, 0.94-1.13) in arm 6 compared with the reference group (arm 7), with a base rate of 4131 of 8448 (48.9%) (Table 3). A shorter-term measure of adherence (defined as PDC measured from the date of enrollment through the end of 2023 rather than for the full year) also did not differ significantly across treatment arms; compared with a rate of 4566 of 5029 (90.8%) in the reference group (arm 7), the odds ratios for adherence were 0.95 (95% CI, 0.77-1.17) in arm 1, 1.06 (95% CI, 0.86-1.32) in arm 2, 0.99 (95% CI, 0.80-1.22) in arm 3, 0.99 (95% CI, 0.80-1.23) in arm 4, 0.99 (95% CI, 0.80-1.22) in arm 5, and 1.00 (95% CI, 0.81-1.24) in arm 6 (Table 3). Effects across all secondary analyses were not statistically significant.

**Table 3.** Odds of Medication Adherence Associated With Intervention

**Outcome**	**Arm 1: baseline mailing**		**Arm 2: baseline plus dynamic social norm**		**Arm 3: baseline plus messenger effects**		**Arm 4: processing fluency**		**Arm 5: processing fluency plus dynamic social norm**		**Arm 6: processing fluency plus messenger effects**		**Arm 7: control (no mailing)**	
	**No. (%)**	**OR (95% CI)**	**No. (%)**	**OR (95% CI)**	**No. (%)**	**OR (95% CI)**	**No. (%)**	**OR (95% CI)**	**No. (%)**	**OR (95% CI)**	**No. (%)**	**OR (95% CI)**	**No. (%)**	**OR (95% CI)**
Hypertension PDC ≥80% after intervention	5039 (90.3)	0.95 (0.77-1.17)	5094 (91.3)	1.06 (0.86-1.32)	4930 (90.7)	0.99 (0.80-1.22)	4943 (90.8)	0.99 (0.80-1.23)	4960 (90.7)	0.99 (0.80-1.22)	5050 (90.8)	1.00 (0.81-1.24)	5029 (90.8)	1 [Reference]
Refill by last day of medication on hand	8471 (48.5)	0.98 (0.90-1.08)	8454 (50.3)	1.06 (0.96-1.16)	8455 (49.5)	1.02 (0.93-1.12)	8456 (49.3)	1.02 (0.93-1.12)	8439 (48.7)	0.99 (0.90-1.09)	8442 (49.7)	1.03 (0.94-1.13)	8448 (48.9)	1 [Reference]

Abbreviations: OR, odds ratio; PDC, proportion of days covered.

Subgroup analyses are shown in Table 4. These results were similar to the overall findings in Table 2 and Table 3, with no differences across subgroups.

**Table 4.** Changes in Medication Adherence Associated With Intervention, by Subpopulation^a^

Subgroup	Arm 1: baseline mailing		Arm 2: baseline plus dynamic social norm		Arm 3: baseline plus messenger effects		Arm 4: processing fluency		Arm 5: processing fluency plus dynamic social norm		Arm 6: processing fluency plus messenger effects	
	Absolute difference (95% CI)	*P* value	Absolute difference (95% CI)	*P* value	Absolute difference (95% CI)	*P* value	Absolute difference (95% CI)	*P* value	Absolute difference (95% CI)	*P* value	Absolute difference (95% CI)	*P* value
Age, y												
65-69	−0.02 (−0.04 to 0.00)	.18	−0.02 (−0.04 to 0.00)	.11	−0.04 (−0.06 to 0.01)	.04	−0.02 (−0.04 to 0.00)	.05	−0.02 (−0.04 to 0.00)	.04	−0.01 (−0.03 to 0.01)	.17
70-74	0.01 (−0.02 to 0.03)		0.01 (−0.01 to 0.04)		0.00 (−0.02 to 0.03)		0.01 (−0.01 to 0.04)		0.02 (−0.01 to 0.04)		0.02 (0.00 to 0.04)	
75-80	−0.01 (−0.03 to 0.02)		0.01 (−0.02 to 0.03)		0.00 (−0.03 to 0.02)		−0.02 (−0.04 to 0.01)		0.00 (−0.03 to 0.02)		0.00 (−0.02 to 0.03)	
Sex												
Female	−0.01 (−0.03 to 0.01)	.89	−0.01 (−0.02 to 0.01)	.31	−0.01 (−0.03 to 0.01)	.87	−0.02 (−0.03 to 0.00)	.19	−0.01 (−0.03 to 0.01)	.15	0.00 (−0.02 to 0.01)	.37
Male	−0.01 (−0.03 to 0.01)		0.01 (−0.01 to 0.03)		−0.01 (−0.03 to 0.01)		0.00 (−0.02 to 0.02)		0.01 (−0.01 to 0.03)		0.01 (−0.01 to 0.03)	
Baseline adherence^b^												
Low	−0.01 (−0.03 to 0.01)	.73	0.01 (−0.01 to 0.03)	.49	−0.02 (−0.04 to 0.00)	.52	−0.01 (−0.03 to 0.01)	.66	0.00 (−0.02 to 0.02)	.89	0.01 (−0.01 to 0.03)	.78
High	−0.01 (−0.03 to 0.01)		0.00 (−0.02 to 0.01)		−0.01 (−0.02 to 0.01)		0.00 (−0.02 to 0.01)		0.00 (−0.01 to 0.02)		0.00 (−0.02 to 0.02)	
Eligible for LIS												
Yes	−0.01 (−0.03 to 0.02)	.73	0.00 (−0.02 to 0.03)	.69	−0.01 (−0.04 to 0.01)	.80	0.00 (−0.03 to 0.02)	.59	−0.01 (−0.03 to 0.01)	.50	0.00 (−0.02 to 0.03)	.97
No	−0.01 (−0.03 to 0.01)		0.00 (−0.02 to 0.01)		−0.01 (−0.03 to 0.00)		−0.01 (−0.03 to 0.01)		0.00 (−0.01 to 0.02)		0.00 (−0.01 to 0.02)	
Baseline refill cycle, d												
30	−0.01 (−0.04 to 0.02)	.97	0.01 (−0.02 to 0.04)	.40	0.00 (−0.03 to 0.03)	.35	0.00 (−0.03 to 0.03)	.34	0.00 (−0.03 to 0.03)	.66	0.01 (−0.02 to 0.04)	.54
90	−0.01 (−0.01 to 0.01)		0.00 (0.00 to 0.00		−0.02 (−0.02 to 0.02)		−0.01 (−0.01 to 0.01)		0.00 (0.00 to 0.00)		0.00 (0.00 to 0.00)	

Abbreviation: LIS, low-income subsidy.

^a^ Reference group was arm 7 (usual care, no mailing). *P* values are for interaction.

^b^ Low adherence was defined as less than 78% of the proportion of days covered and high as 78% or more.

## Discussion

In this large, 7-arm, open-label randomized clinical trial, there were no statistically significant differences in hypertension medication adherence between treatment and control groups. Social norming effects have previously been shown to be effective for a variety of health improvement outcomes, including smokers’ intention to quit, dietary choice, sleep-related behavior, and reducing red meat consumption.^8,9^ In addition, messenger effects in the form of pharmacist text messages have been shown to be associated with improved hypertensive medication adherence.^19^ Furthermore, measures can be incorporated to make it more attractive to process a message through tactics such as visual metaphors, like use of reference lines denoting speed to improve messages aimed at reducing railroad accidents, especially when low health literacy is a key barrier to attaining health outcomes.^11,20^ In contrast, to our knowledge, these behavioral science principles have not been studied rigorously in the context of a mailing to participants to improve hypertension medication adherence scores.

Our results are similar to those of other studies indicating that summary information on medication adherence—for example, for statin medication adherence—may have null or limited effects on improving medication adherence scores.^15,16^ There are a number of possibilities that may explain the null results in the current study. First, there was no guarantee that participants received, opened, or understood their scorecards. Medication adherence requires many repeated behaviors over the course of an extended period. To measurably change this pattern of behavior, mailed communications may not be salient or intensive enough to create behavior change, and other communication strategies, such as telephone calls or in-person interactions, should be considered. In addition, communications may need to come from multiple and/or different sources, such as the combination of one’s insurance provider, physician, and pharmacist. Second, the intervention was launched in the second half of the year and with limited time to build awareness or change behavior. The end-of-year PDC measurement was calculated based on the full calendar year, including 8 months of behavior before the intervention was launched. Given the volume of study participants with 90-day refill cycles (approximately 3 months), these participants would have had limited opportunity to refill their medications during the 4-month follow-up period. Third, hypertension is asymptomatic, and participants may not be motivated to change adherence patterns based on lack of symptoms. Fourth, medication adherence for hypertension may have ceiling effects: in 2023, approximately 89% of Humana beneficiaries were adherent to their prescribed medications. The remaining 11% were likely the beneficiaries who faced the most challenging barriers to adherence, such as housing instability, which may require more intensive interventions.

### Limitations

Our analysis has several limitations. First, our medication adherence outcomes were calculated based on medication refills, and we were unable to observe whether participants actually took their medications once refilled. Second, as with most medication adherence work conducted through insurance plans, our refill measure was determined based on claims data and did not include any refills not made through the participant’s Humana coverage.

## Conclusions

In this randomized clinical trial, use of social norms, messenger effects, and processing fluency in mailed communications did not significantly increase hypertension medication adherence. However, there are several promising avenues for future research. Better design of communication materials, varying the mode of administration (eg, using a telephonic or digital intervention),^21^ or increasing outreach frequency or duration may have a stronger impact. Future research could also explore alternative methods of outreach with primary care clinicians over longer periods, with coaching support.

## Notes

Supplement 1.
Trial Protocol

Supplement 2. **eFigure.** Participant Enrollment by Study Wave

Supplement 3.
Data Sharing Statement
