# Supplementary material for: Incorporating Behavioral Science in Medication Adherence Communication: A Randomized Clinical Trial
Source: JAMA Netw Open. 2025 May 14;8(5):e2510162. doi: 10.1001/jamanetworkopen.2025.10162 (PMC12079288; doi:10.1001/jamanetworkopen.2025.10162)
Supplement: Supplement 1. — Trial Protocol [file jamanetwopen-e2510162-s001.pdf]

# CLINICAL TRIAL PROTOCOL

Social norms, messengers, and processing fluency to increase hypertension medication adherence

**Principal Investigator:**
Linda Chung, PharmD, MBA, BCACP
Clinical Lead, Humana, Inc.

**Version 1.3**
July 28, 2023

# Table of Contents


PRÉCIS ........ 4

PARTICIPATING STUDY SITES ........ 6

1 Study objectives ........ 6

1.1 Primary Objective ........ 6

1.2 Secondary Objectives ........ 6

2 BACKGROUND AND RATIONALE ........ 6

2.1 Background on Condition, Disease, or Other Primary Study Focus ........ 6

2.2 Study Rationale 6

3 STUDY DESIGN ........ 7

4 SELECTION AND ENROLLMENT OF PARTICIPANTS ........ 9

4.1 Inclusion Criteria ........ 9

4.2 Exclusion Criteria ........ 9

4.3 Study Enrollment Procedures ........ 9

5 STUDY INTERVENTIONS ........ 10

5.1 Interventions, Administration, and Duration ........ 10

5.2 Handling of Study Interventions ........ 11

5.3 Concomitant Interventions ........ 11

5.3.1 Allowed Interventions ........ 11

5.3.2 Required Interventions ........ 11

5.3.3 Prohibited Interventions ........ 11

5.4 Adherence Assessment ........ 11

6 STUDY PROCEDURES ........ 11

6.1 Schedule of Evaluations ........ 11

6.2 Description of Evaluations ........ 12

6.2.1 Screening Evaluation ........ 12

6.2.2 Enrollment, Baseline, and/or Randomization ........ 12

6.2.3 Follow-up Visits ........ 12

6.2.4 Completion/Final Evaluation ........ 12

*7* SAFETY ASSESSMENTS ........ 12

7.1 Specification of Safety Parameters ........ 12

7.2 Methods and Timing for Assessing, Recording, and Analyzing Safety Parameters ........ 13

7.3 Adverse Events and Serious Adverse Events ........ 13

7.3.1 Reporting Procedures....13
7.3.2 Follow-up for Adverse Events....13

8 INTERVENTION DISCONTINUATION.... 14

9 STATISTICAL CONSIDERATIONS.... 14

9.1 General Design Issues.... 14

9.2 Sample Size and Randomization.... 14
9.2.1 Treatment Assignment Procedures....14

9.3 Interim analyses and Stopping Rules.... 15

9.4 Outcomes 15
9.4.1 Primary outcome....15
9.4.2 Secondary outcomes....15

9.5 Data Analyses 15

10 DATA COLLECTION AND QUALITY ASSURANCE.... 15

10.1 Data Collection Forms.... 15

10.2 Data Management.... 15

10.3 Quality Assurance.... 16
10.3.1 Training....16
10.3.2 Quality Control Committee....16
10.3.3 Metrics....16
10.3.4 Protocol Deviations....16
10.3.5 Monitoring....16

11 PARTICIPANT RIGHTS AND CONFIDENTIALITY.... 16

11.1 Institutional Review Board (IRB) Review.... 16

11.2 Informed Consent Forms.... 16

11.3 Participant Confidentiality.... 17

11.4 Study Discontinuation.... 17

12 ETHICAL CONSIDERATIONS.... 17

13 REFERENCES.... 19

## PRÉCIS

### Study Title

Social norms, messengers, and processing fluency to increase hypertension medication adherence

### Objectives

Aim 1: Test the effect of dynamic social norms, messenger effects, and processing fluency messages on hypertension medication adherence. The study team will identify 59,997 Medicare Advantage beneficiaries insured by Humana who are not fully adherent to hypertension medications and randomize them to one of six mailed communications to increase medication adherence or control. The primary outcome will be medication adherence (measured by pharmacy refill claims).

Aim 2: Assess association of claims and demographic characteristics with intervention responsiveness. The study team will evaluate the impact of the interventions as a function of baseline levels of adherence, demographic characteristics, and clinical comorbidities to determine the association of patient characteristics with intervention responsiveness. These profiles could be used to target future message content and framing to specific patients most likely to respond.

### Design and Outcomes

Design: randomized controlled clinical trial of patient-facing messages to test the impact of social norms, messengers, and processing fluency on hypertension medication adherence in individuals 65 to 79 years of age taking medication for hypertension with adherence levels (based on the proportion of days covered metric [PDC]) between 60% and 85%.

The primary outcome will be the average end-of-year PDC in each arm. A secondary outcome will be the proportion of study participants with end-of-year PDC≥80%.

See **Figure 1** for an overview of the study design.

*Figure 1: Overview of Study Design*

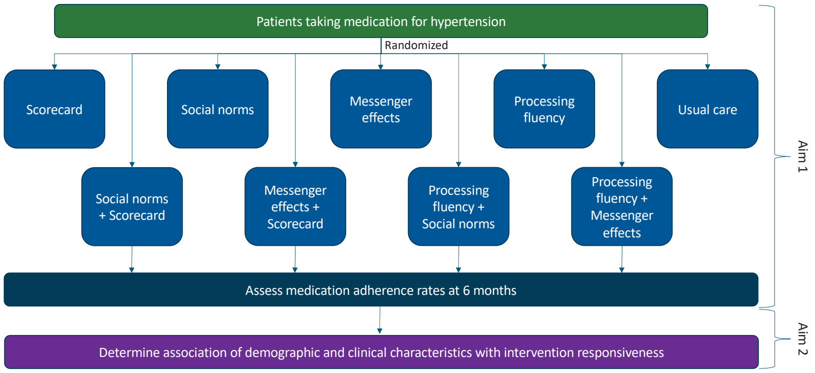


### Interventions and Duration

Study participants will be randomly assigned to one of seven conditions:

1. A scorecard from Humana reporting patients' medication adherence using a "refill score"
2. The Arm 1 scorecard plus dynamic social norms messaging (noting the proportion of Humana members improving their medication refill scores)
3. The Arm 1 scorecard, coming from the trusted messenger of a Humana-identified pharmacist taking the same medication

4. A modified scorecard increasing processing fluency through a visual metaphor of "closing the ring"
5. The Arm 4 scorecard plus dynamic social norms messaging
6. The Arm 4 scorecard, coming from the trusted messenger of a Humana-identified pharmacist taking the same medication
7. No mailed message (usual care)

Participants will participate in this study for approximately 6 months (through December 2023).

**Sample Size and Population**

Target population: 59,997 Humana Medicare patients 65 to 79 years of age taking hypertension medications with adherence level (based on the proportion of days covered metric [PDC]) between 60% and 85%. Divided across 7 arms, there will be 8,571 participants in each arm. Randomization will be stratified by whether participants are on a 30-day medication refill cycle (~26% of overall study population) or a 90-day medication refill cycle (~74% of overall study population).

## STUDY TEAM ROSTER

**Principal Investigator:** **Linda Chung, PharmD, MBA, BCACP**
Humana, Inc.
500 W. Main Street
Louisville, KY 40202
Tel: 949-491-5242
Lchung1@humana.com

**Main responsibilities/Key roles:** Responsible for overseeing the trial execution and evaluation activities; oversight of activities related to the trial execution and evaluation of the effectiveness of the interventions; overseeing the activities of the sub-investigators and study staff.

## PARTICIPATING STUDY SITES

Study participants will be selected from Humana, a large insurance provider.

# 1 STUDY OBJECTIVES

## 1.1 Primary Objective

Hypothesis: Messages using dynamic social norms, messenger effects, and processing fluency in combination will more effectively increase average end-of-year adherence level compared to usual care.

## 1.2 Secondary Objectives

Secondary objective: identify baseline clinical and demographic characteristics to predict the efficacy of each message.

# 2 BACKGROUND AND RATIONALE

## 2.1 Background on Condition, Disease, or Other Primary Study Focus

Hypertension substantially increases the risk of myocardial infarction and stroke,[1] but medications that can effectively control hypertension (are not always taken as prescribed: adherence rates for hypertension medications range from about 40% to 74% for most Americans.[2-4] Non-adherence is therefore a potent contributor to preventable disability and death among middle-aged and older adults.[5] There also exist disparities by race and ethnicity among Medicare Part D beneficiaries, Black and Hispanic patients are 38% and 45% more likely, respectively, to be nonadherent compared to white patients.[6]

## 2.2 Study Rationale

Interventions to improve medication adherence have had modest success;[7,8] even the most effective interventions combining behavioral, educational, and logistical interventions for providers, health systems, and patients have limited effects.[9-11] Leveraging behavioral principles of social norms, messenger effects, and processing fluency may improve their impact.

Social norms describe perceptions about the behavior of others, which can influence one's own behavior. Making information about others' behavior more salient can encourage those behaviors when base rates are high. When they are low, dynamic norms, or information about how others' behavior is *changing*, may be more impactful.[12] Dynamic norms have shown impact in a range of healthy behaviors.[13]

The effectiveness of a message also often depends on who is communicating it. Messenger impact can differ depending on factors such as how trusted they are, or how much they have in common with the people they are communicating with. Messenger effects have been successfully leveraged in HIV prevention and management[14] and diabetes screening.[15] Leveraging pharmacists in hypertension medication adherence interventions has shown promise, mainly in direct phone calls from pharmacists.[16,17] Having medication-related messaging come from an insurance company's pharmacist rather than the insurance company itself may increase its impact, especially with pharmacists who are taking the same medications, and with lower cost through written communication rather than a phone call.

Processing fluency is the subjective ease with which information is understood, which can influence its actionability. Increasing processing fluency has been impactful for exercise[18] and other health behaviors,[19] including treatment adherence for juvenile idiopathic arthritis[20] and cancer screening.[21] Studies providing patients with summary information for statin medication adherence have demonstrated null[22] or limited[23] results, but incorporating measures to ease processing fluency through tactics such as visual metaphors that emphasize goal progress and attainability may increase their impact, especially as low health literacy is a key barrier to medication adherence.[24]

Messages may have varied effectiveness based on patients' level of adherence and/or demographic factors, which are also associated with differences in adherence.[4,6] Tailoring a message based upon these factors could optimize future intervention effectiveness.

Thus, the study team proposes a randomized trial to test the effects of mailed communication leveraging social norms, messenger effects, and processing fluency on hypertension medication adherence for patients who are not fully adherent, followed by analyses identifying intervention responsiveness by patient characteristics.

## 3 STUDY DESIGN

**Type/design of trial:** randomized controlled trial

**Primary objective (hypothesis):** Messages using dynamic social norms, messenger effects, and processing fluency in combination will more effectively increase average end-of-year adherence level compared to usual care (no messages).

**Secondary objective:** To identify baseline clinical and demographic characteristics to predict the efficacy of each message.

**Study population:** Individuals 65 to 79 years of age taking any class of medication for hypertension, with adherence level between 60% and 85%.

**Study arms (7,143 individuals each):**

1. A scorecard from Humana reporting patients’ medication adherence using a “refill score”
2. The Arm 1 scorecard plus dynamic social norms messaging (noting the proportion of Humana members improving their medication refill scores)
3. The Arm 1 scorecard, coming from the trusted messenger of a Humana-identified pharmacist taking the same medication.
4. A modified scorecard increasing processing fluency through a visual metaphor of “closing the ring”
5. The Arm 4 scorecard plus dynamic social norms messaging
6. The Arm 4 scorecard, coming from the trusted messenger of a Humana-identified pharmacist taking the same medication.
7. No mailed message (usual care)

**Study location:** Humana

**Duration of enrollment period:** Individuals will be enrolled at a single time point at study launch

**Duration of follow-up period:** Approximately 6 months

**Description of intervention and administration:** Study participants will be randomized into receiving one of 6 mailed messages (arms 1-6 above) informing them of their medication refill score, or no message. Two messages will be sent during the intervention: The first message will contain the baseline refill score. The second message will note any changes in the refill score.

**Description of descriptive and outcome variables from the medical, insurance, and pharmaceutical records:**

Medical variables: None

Insurance / enrollment variables:

- Patient enrollment in a Medicare Advantage Prescription Drug plan insured by Humana in plan year 2023
- Patient age in plan year 2023
- Patient Gender
- Patient Race

Pharmaceutical variables:

- Current adherence on hypertension medications (PDC) as of July 2023
- Unique prescription fills for hypertension medication use
- Unique prescription fills for other medications
- End-of-year adherence on hypertension medications (PDC) in 2023 (following intervention)

## 4 SELECTION AND ENROLLMENT OF PARTICIPANTS

### 4.1 Inclusion Criteria

Patients must meet all inclusion criteria to participate in this study:

- Medicare Advantage beneficiary insured by Humana
- Between the ages of 65 and 79
- Having at least two unique fills of any class of hypertension medication within the calendar year
- Adherence level (as measured by the proportion of days covered [PDC] metric) between 60% and 85%

### 4.2 Exclusion Criteria

Humana policy specifies that certain subgroups of members should not be included in research studies per contractual agreements, privacy policies and rules, or legal requirements. These excluded entities and groups should not be considered part of the overall Humana Research Population. Members that meet the following criteria will be excluded:

- Commercial Fully Insured Groups to be excluded from Research (These are actually employer groups that provide Medicare Group coverage to their retired employees). Each quarter Data Offerings revises and updates the file of excluded Commercial Groups.
    a. Blue Cross Blue Shield of Florida
    b. Employees Retirement System of Texas,
    c. Georgia Department of Community Health
    d. Ohio Operating Engineers
    e. Ohio Public Employees Retirement System
    f. School Board of Broward County
    g. Public Employees Insurance Agency, an agency of the State of West Virginia,
    h. Missouri Blind Pension Program
- Florida Medicaid members
- Illinois MMAI members
- Puerto Rico state of residence and from any other geographical areas that are outside of the 50 states (residents of the District of Columbia are not excluded). A few members have residence in the Virgin Islands or other US territories (i.e. Guam, American Samoa). They constitute a small count of patients and cannot be assigned to the 4 geographical areas typically reported for research.
- Administrative Services Only (ASO)
- All Group Medicare members

### 4.3 Study Enrollment Procedures

For this study, potential participants will be identified through Humana-based datasets. The interventions in this study will be integrated in the usual patient messaging workflow. All Medicare Advantage beneficiaries have provided addresses to Humana

that can be used for mailed communications. 59,997 Medicare Advantage beneficiaries insured by Humana who are not fully adherent to hypertension medications, defined as patients with adherence level (as measured by the Proportion of Days Covered metric [PDC)) between 60% and 85% (i.e., a "refill score" between 60% and 85%).

Study arms include six intervention arms and a usual care arm. All interventions will specifically target hypertension medication adherence. All participants in the six intervention arms will receive two mailed letters. As with other minimal-risk, quality improvement studies the study team has performed that involve the use of routine clinical tools such as patient portals and reminder letters and where informed consent is impracticable, the study team is requesting a waiver of informed consent and HIPAA authorization. There are several reasons for this. One, the nature of this quality improvement intervention involves testing different framing types in patient letters using a similar infrastructure as used during regular insurance communications. Second, the ability to understand the true effect of the intervention as it is delivered in the real world would be difficult to ascertain if formal informed consent from patients were sought. Third, obtaining formal informed consent would predictably reduce the number of patients participating in the study, especially those from under-represented populations, and therefore undermine the generalizability of the study results, a foundational aspect of pragmatic clinical trial principles. In our prior work, the study team has received a waiver of informed consent from the IRB for similar interventions.

# 5 STUDY INTERVENTIONS

## 5.1 Interventions, Administration, and Duration

Study arms:

1. A scorecard from Humana reporting patients' medication adherence using a "refill score"
2. The Arm 1 scorecard plus dynamic social norms messaging (noting the proportion of Humana members improving their medication refill scores)
3. The Arm 1 scorecard, coming from the trusted messenger of a Humana-identified pharmacist taking the same medication
4. A modified scorecard increasing processing fluency through a visual metaphor of "closing the ring"
5. The Arm 4 scorecard plus dynamic social norms messaging
6. The Arm 4 scorecard, coming from the trusted messenger of a Humana-identified pharmacist taking the same medication
7. No mailed message (usual care)

Study participants will be randomized into one of 7 arms. Participants in a treatment arm (arms 1-6 above) will receive 1 of 6 variations of a mailed message informing them of their medication refill score. Participants in the usual care arm will not receive a message. All participants in the six intervention arms will receive two mailed letters in total. The first message will contain the baseline refill score. The second message will be sent within 60 days of the first message and note any changes in the refill score.

## 5.2 Handling of Study Interventions

Quality control will be completed and messages will be sent by Humana's internal communications teams, who have extensive experience sending similar messages and conducting similar studies. All messages will have similar overall design and language, with the exception of specific sections of the messages that contain the behavioral elements to be tested.

## 5.3 Concomitant Interventions

None.

### 5.3.1 Allowed Interventions

None.

### 5.3.2 Required Interventions

None.

### 5.3.3 Prohibited Interventions

None.

## 5.4 Adherence Assessment

Not applicable—no compliance requirements in the study.

# 6 STUDY PROCEDURES

## 6.1 Schedule of Evaluations

| Assessment | Baseline, Enrollment, Randomization: (Day 0) | Treatment 1 (Day 1) | Treatment 2 (Day 53) | Final analysis |
|---|---|---|---|---|
| *Identification of eligible patients* | X | | | |
| *Enrollment/Randomization* | X | | | |
| *Intervention (control and experimental)* | | X | X | |
| *Outcome analysis* | | | | X |

## 6.2 Description of Evaluations

### 6.2.1 Screening Evaluation

Consenting Procedure

As with other minimal-risk, quality improvement studies the study team has performed that involve the use of routine clinical tools such as patient portals and reminder letters, formal informed consent will not be sought.

Screening

Eligibility decisions will be decided based on age and adherence level in July 2023.

### 6.2.2 Enrollment, Baseline, and/or Randomization

Enrollment

59,997 randomly selected study participants will be enrolled from the pool of Humana members who meet all study inclusion criteria and none of the study exclusion criteria.

Baseline Assessments

- Current adherence level as of July 2023.
- Historical, and demographic patient characteristics including age, gender, race, clinical comorbidities and other medication use

Randomization

All study participants will be randomized post-screening and pre-initiation of study intervention.

### 6.2.3 Follow-up Visits

Not applicable (there are no follow-up visits included in this study).

### 6.2.4 Completion/Final Evaluation

Not applicable (there are no follow-up visits included in this study).

# *7* SAFETY ASSESSMENTS

## 7.1 Specification of Safety Parameters

**The study team believes that the risks to participation for members are no more than minimal for several reasons.** First, the interventions encourage the refill of hypertension medications indicated for and prescribed by patients' physicians. Second, the study team will not be providing any direct care to patients, and all hypertension medication refill decisions will ultimately be made by the patient, who will have the option of contacting their medical team and/or Humana for advice or specific recommendations. To minimize the risks to privacy from use of patient data, the study team will safeguard any identifiable information and limit access to the information to study investigator actively involved in the research who have all undergone human subjects research training, using a study ID in all other circumstances.

In this pragmatic trial, the study team does not anticipate the occurrence of any incremental adverse events as a result of patients receiving information that encourages them to refill their prescribed hypertension medications on time. All patients involved in the study have been prescribed these medications, and the study team will exclude patients with refill scores that are already ≥85%. Any adverse events from the medications themselves will be handled in the course of regular clinical care, as they are currently.

## 7.2 Methods and Timing for Assessing, Recording, and Analyzing Safety Parameters

Not applicable due to the minimal risk profile of the proposed study.

## 7.3 Adverse Events and Serious Adverse Events

*Adverse Event (AE):* Any untoward or unfavorable medical occurrence in a human study participant, including any abnormal sign (e.g., abnormal physical exam or laboratory finding), symptom, or disease, temporally associated with the participants' involvement in the research, whether or not considered related to participation in the research.

*Serious Adverse Event (SAE):* Any adverse event that:
- Results in death
- Is life threatening, or places the participant at immediate risk of death from the event as it occurred
- Requires or prolongs hospitalization
- Causes persistent or significant disability or incapacity
- Results in congenital anomalies or birth defects
- Is another condition which investigators judge to represent significant hazards

**Given the minimal risk nature of the study, in which the intervention involves enhanced communication about hypertension medication adherence for Humana Medicare Advantage beneficiaries for quality improvement purposes, the study team does not expect any SAEs or AEs related to the messages delivered in this trial.**

### 7.3.1 Reporting Procedures

No additional SAEs or AEs are expected as expected to take place as a result of participation in this study, as the study team will not be providing any direct care to members and all hypertension medication adherence decisions will ultimately be made by the patient, with the potential consultation of their medical team. If the study team becomes aware of any AEs or SAEs throughout the course of the study, the study team will collect this information.

AE/SAE reporting to the IRB of record will also be completed in compliance with IRB reporting standards.

### 7.3.2 Follow-up for Adverse Events

No additional SAEs or AEs are expected to take place as a result of participation in this study, as the study team will not be providing any direct care to members and all hypertension medication adherence decisions will ultimately be made by the patient, with the potential consultation of their medical team. However, the study team will be

notified if patients reach out to Humana with feedback about the intervention messages. If the study team becomes aware of any AEs or SAEs throughout the course of the study, the study team will collect and document this information. All AEs will be followed to adequate resolution.

Any medical condition that is present at the time that the participant is screened will be considered as baseline and not reported as an AE. However, if the study participant's condition deteriorates at any time during the study, it will be recorded as an AE.

Changes in the severity of an AE will be documented to allow an assessment of the duration of the event at each level of severity to be performed. AEs characterized as intermittent require documentation of onset and duration of each episode.

Events will be followed for outcome information until resolution or stabilization.

# 8 INTERVENTION DISCONTINUATION

Not applicable—due to minimal risk of proposed study, there are no discontinuation criteria.

# 9 STATISTICAL CONSIDERATIONS

## 9.1 General Design Issues

The study team hypothesizes that messages using dynamic social norms, messenger effects, and processing fluency in combination will more effectively increase adherence compared to usual care, and can identify baseline clinical and demographic characteristics to predict the efficacy of each message.

The primary outcome will be the average end-of-year adherence level in each arm, stratified by refill cycle. The study team will use Humana claims data for outcome measurement, which contains information on prescription claims from pharmacies, as well as patient information. Linear regression will be used to compare outcomes between each intervention arm versus usual care.

## 9.2 Sample Size and Randomization

The study team estimates a sample size of 59,997 (8,571 per arm) will provide 80% power to observe a ~1% difference in end-of-year adherence level compared to the usual care arm, conservatively assuming a usual care arm rate of 70% and a Holm-Bonferroni corrected alpha of 0.05 to adjust the familywise error rates for multiple hypotheses testing. This difference is significant as the focus of CMS Star ratings measure interventions and are comparable to improvements seen in other similar large-scale behavioral communication studies. In secondary analyses, the study team will compare arms against each other and adjust for any imbalanced baseline characteristics.

### 9.2.1 Treatment Assignment Procedures

Study participants will be randomized in a 1:1:1:1:1:1:1 ratio with a random number generator into one of 7 arms, stratified by whether participants are on a 30-day

medication refill cycle (~26% of overall study population) or a 90-day medication refill cycle (~74% of overall study population).

### 9.3 Interim analyses and Stopping Rules

Not applicable—interim analyses are not planned.

### 9.4 Outcomes

#### 9.4.1 Primary outcome

The primary outcome will be the average end-of-year adherence level in each arm, stratified by refill cycle and adjusting for age, sex, and race.

#### 9.4.2 Secondary outcomes

The secondary outcome will be the proportion of study participants with end-of-year PDC≥80%.

In secondary analyses, the study team will compare arms against each other and adjust for any imbalanced baseline characteristics.

### 9.5 Data Analyses

The study team will use Humana claims data for outcome measurement, which contains information on prescription claims from pharmacies, as well as patient information. Regular linear regression will be used to compare outcomes between each intervention arm versus usual care adjusting for the stratified randomized design, age, gender, and race. These adjustments are necessary as the baseline differences in adherence in each of these groups could influence their responses to our intervention.

## 10 DATA COLLECTION AND QUALITY ASSURANCE

### 10.1 Data Collection Forms

The study team will use the Humana claims data for outcome measurement, which contains information on prescription claims from pharmacies, as well as patient information.

### 10.2 Data Management

Individually identifiable data are maintained for administrative purposes at Humana and are needed to identify members to contact for the intervention. Without use of individual level data, the study team could not fulfill the study’s objectives. To protect the confidentiality of these data, only Humana associates with the appropriate data privileges will have access to personal identifiers while linking the data and constructing study variables for the trial.

## 10.3 Quality Assurance

### 10.3.1 Training

All members of the research team have completed or will complete appropriate human subjects research training and patient privacy training related to the Health Insurance Portability and Accountability Act (HIPAA).

### 10.3.2 Quality Control Committee

Not applicable—no study quality control committee.

### 10.3.3 Metrics

Quality control metrics for outcome measures will include checks that messages are being a) correctly received based on randomization assignment and b) correctly targeted to 30- vs. 90-day refill cycles. The study team will conduct random checks that targeting is being done correctly.

### 10.3.4 Protocol Deviations

The principal investigator will review study conduct (e.g., protocol deviations) on a monthly basis. The principal investigator will also ensure that all protocol deviations for the trials are reported to the IRB according to the applicable regulatory requirements.

### 10.3.5 Monitoring

The study team will be notified if patients or staff reach out to Humana with feedback about the intervention messages. If the study team becomes aware of any AEs or SAEs throughout the course of the study, the study team will collect this information. Any reports of deaths will be submitted to the IRB within 24 hours. Any unexpected SAEs will be reported to the IRB within 48 hours of the study's knowledge of the SAE. All other reported SAEs and AEs received by the study team will be reported to the IRB quarterly.

# 11 PARTICIPANT RIGHTS AND CONFIDENTIALITY

## 11.1 Institutional Review Board (IRB) Review

This protocol and any subsequent modifications will be reviewed and approved by the IRB responsible for oversight of the study.

## 11.2 Informed Consent Forms

As with other minimal-risk, quality improvement studies the study team has performed that involve the use of routine clinical tools such as patient portals and reminder letters and where informed consent is impracticable, the study team is requesting a waiver of informed consent and HIPAA authorization. There are several reasons for this. One, the nature of this quality improvement intervention involves testing different framing types in patient letters using a similar infrastructure as used during regular insurance communications. Second, the ability to understand the true effect of the intervention as it is delivered in the real world

would be difficult to ascertain if formal informed consent from patients were sought. Third, obtaining formal informed consent would predictably reduce the number of patients participating in the study, especially those from under-represented populations, and therefore undermine the generalizability of the study results, a foundational aspect of pragmatic clinical trial principles. The study team has received a waiver of informed consent from the IRB for similar interventions.

### 11.3 Participant Confidentiality

Individually identifiable data are maintained for administrative purposes at Humana and are needed to identify members to contact for the intervention. Without use of individual level data, the study team could not fulfill the study's objectives. To protect the confidentiality of these data, only Humana associates with the appropriate data privileges, will have access to personal identifiers while linking the data and constructing study variables for the trial. All data will be maintained at Humana, where the IRB-approved analyses will be performed. Only aggregated study results will be shared externally. All study team members are trained in research management.

To protect against the risk of inappropriate disclosure of personal health information, no identifiable or individual level data will be shared outside of Humana and all data will be stored securely, as they are at present. All members of the research team have completed or will complete appropriate human subjects research training and patient privacy training related to HIPAA.

To protect against the risk of inappropriate disclosure of personal health information, only Humana team members will access study data with encrypted identifiers. As described, all members of the research team have completed or will complete appropriate human subjects research training and patient privacy training related to the Health Insurance Portability and Accountability Act (HIPAA).

### 11.4 Study Discontinuation

The study may be discontinued at any time by the IRB, the OHRP, the FDA, or other government agencies as part of their duties to ensure that research participants are protected.

## 12 ETHICAL CONSIDERATIONS

General oversight of the project by the principal investigator will occur throughout the study period, including regular contact with the study team to obtain ongoing feedback.

The study team will not be providing any direct care to patients, and all medication decisions will ultimately be made by the patient, with the potential consultation of their medical team. Any adverse events will be handled in the course of regular clinical care. Given the minimal risks involved in participation in this study, the study team does not anticipate any unacceptable adverse events. However, our plan for data and safety

monitoring does include multiple mechanisms to ensure minimal risk of participation in the research.

If the study team becomes aware of any AEs or SAEs throughout the course of the study, the study team will collect this information. Any reports of deaths will be submitted to the IRB within 24 hours. Any unexpected SAEs will be reported to the IRB within 48 hours of the study's knowledge of the SAE. All other reported SAEs and AEs received by the study team will be reported to the IRB quarterly.

## 13 REFERENCES


1. Centers for Disease Control and Prevention, National Center for Health Statistics. About Multiple Cause of Death, 1999–2020. CDC WONDER Online Database website. Atlanta, GA: Centers for Disease Control and Prevention; 2022. Accessed March 1, 2023.
2. Benjamin EJ, Blaha MJ, Chiuve SE, Cushman M, Das SR, Deo R, et al. ; American Heart Association Statistics Committee and Stroke Statistics Subcommittee. Heart disease and stroke statistics — 2017 update: a report from the American Heart Association. Circulation 2017;135(10):e146–603. Errata in: Circulation 2017; 7;135(10):e146-e603 and Circulation 2017;136(10):e196.
3. Ritchey M, Chang A, Powers C, Loustalot F, Schieb L, Ketcham M, et al. Vital signs: disparities in antihypertensive medication nonadherence among Medicare Part D beneficiaries — United States, 2014. MMWR Morb Mortal Wkly Rep 2016;65(36):967–76.
4. Briesacher BA, Andrade SE, Fouayzi H, Chan KA. Comparison of drug adherence rates among patients with seven different medical conditions. Pharmacotherapy 2008;28(4):437–43.
5. Burnier M, Polychronopoulou E, Wuerzner G. Hypertension and Drug Adherence in the Elderly. Front Cardiovasc Med. 2020 Apr 7;7:49. doi: 10.3389/fcvm.2020.00049. PMID: 32318584; PMCID: PMC7154079.
6. Holmes HM, Luo R, Hanlon JT, Elting LS, Suarez-Almazor M, Goodwin JS. Ethnic disparities in adherence to antihypertensive medications of medicare part D beneficiaries. J Am Geriatr Soc. 2012 Jul;60(7):1298-303. doi: 10.1111/j.1532-5415.2012.04037.x. Epub 2012 Jun 15. PMID: 22702464; PMCID: PMC3396714.
7. Choudhry NK, Kronish IM, Vongpatanasin W, Ferdinand KC, Pavlik VN, Egan BM, Schoenthaler A, Houston Miller N, Hyman DJ; American Heart Association Council on Hypertension; Council on Cardiovascular and Stroke Nursing; and Council on Clinical Cardiology. Medication Adherence and Blood Pressure Control: A Scientific Statement From the American Heart Association. Hypertension. 2022 Jan;79(1):e1-e14. doi: 10.1161/HYP.0000000000000203. Epub 2021 Oct 7. PMID: 34615363.
8. Kim SJ, Kwon OD, Han EB, Lee CM, Oh SW, Joh HK, Oh B, Kwon H, Cho B, Choi HC. Impact of number of medications and age on adherence to antihypertensive medications: A nationwide population-based study. Medicine (Baltimore). 2019 Dec;98(49):e17825. doi: 10.1097/MD.0000000000017825. PMID: 31804305; PMCID: PMC6919523.
9. McDonald HP, Garg AX, Haynes RB. Interventions to enhance patient adherence to medication prescriptions: scientific review. JAMA. 2002;288(22):2868–2879.
10. Takiya LN, Peterson AM, Finley RS. Meta-analysis of interventions for medication adherence to antihypertensives. Ann Pharmacother. 2004;38(10):1617–1624.

11. Haynes RB, McDonald H, Garg AX, Montague P. Interventions for helping patients to follow prescriptions for medications. The Cochrane Database of Systematic Reviews 2002, Issue 2. Art. No.: CD000011. DOI: 10.1002/14651858.CD000011.
12. Sparkman, G., & Walton, G. M. (2017). Dynamic norms promote sustainable behavior, even if it is counternormative. Psychological science, 28(11), 1663-1674.
13. Sparkman, G., & Walton, G. M. (2019). Witnessing change: Dynamic norms help resolve diverse barriers to personal change. Journal of Experimental Social Psychology, 82, 238-252.
14. Andrawis, A., Tapa, J., Vlaev, I., Read, D., Schmidtke, K.A., Chow, E.P., Lee, D., Fairley, C.K. and Ong, J.J., 2022. Applying behavioural insights to HIV prevention and management: a scoping review. Current HIV/AIDS Reports, 19(5), pp.358-374.
15. Hallsworth, M., Snijders, V., Burd, H., Prestt, J., Judah, G., Huf, S., & Halpern, D. (2016). Applying behavioral insights: simple ways to improve health outcomes. World Innovation Summit for Health, Doha, Qatar, 29–30 November.
16. Santschi, V., Chiolero, A., Colosimo, A.L., Platt, R.W., Taffé, P., Burnier, M., Burnand, B. and Paradis, G., 2014. Improving blood pressure control through pharmacist interventions: a meta-analysis of randomized controlled trials. Journal of the American Heart Association, 3(2), p.e000718.
17. Contreras-Vergara, A., Sifuentes-Franco, S., Haack, S., Graciano-Machuca, O., Rodriguez-Carrizalez, A.D., López-Contreras, A.K., Reyes-Pérez, I.V. and Huerta-Olvera, S.G., 2022. Impact of pharmaceutical education on medication adherence and its clinical efficacy in patients with type 2 diabetes and systemic arterial hypertension. Patient preference and adherence, pp.1999-2007.
18. Song, H., & Schwarz, N. (2008). If it's hard to read, it's hard to do: processing fluency affects effort prediction and motivation. Psychological Science, 19(10), 986-988.
19. Okuhara, T., Ishikawa, H., Okada, M., Kato, M., & Kiuchi, T. (2017). Designing persuasive health materials using processing fluency: a literature review. BMC Research Notes, 10(1), 1-10.
20. Reiser, C., Zeltner, N. A., Rettenbacher, B., Baumgaertner, P., Huemer, M., & Huemer, C. (2021). Explaining juvenile idiopathic arthritis to paediatric patients using illustrations and easy-to-read texts: improvement of disease knowledge and adherence to treatment. Pediatric Rheumatology, 19, 1-10.
21. Okuhara, T., Ishikawa, H., Goto, E., Okada, M., Kato, M., & Kiuchi, T. (2018). Processing fluency effect of a leaflet for breast and cervical cancer screening: A randomized controlled study in Japan. Psychology, Health & Medicine, 23(10), 1250-1260.
22. Reese, P.P., Kessler, J.B., Doshi, J.A., Friedman, J., Mussell, A.S., Carney, C., Zhu, J., Wang, W., Troxel, A., Young, P. and Lawnicki, V., 2016. Two randomized controlled pilot trials of social forces to improve statin adherence among patients with diabetes. Journal of general internal medicine, 31, pp.402-410.

23. Reddy, A., Huseman, T. L., Canamucio, A., Marcus, S. C., Asch, D. A., Volpp, K., & Long, J. A. (2017). Patient and partner feedback reports to improve statin medication adherence: a randomized control trial. Journal of General Internal Medicine, 32, 256-261.

24. Ngoh, L. N. (2009). Health literacy: a barrier to pharmacist–patient communication and medication adherence. Journal of the American Pharmacists Association, 49(5), e132-e149
