# Supplementary material for: Incorporating Behavioral Science in Medication Adherence Communication: A Randomized Clinical Trial
Source: JAMA Netw Open. 2025 May 14;8(5):e2510162. doi: 10.1001/jamanetworkopen.2025.10162 (PMC12079288; doi:10.1001/jamanetworkopen.2025.10162)
Supplement: Supplement 2. — eFigure. Participant Enrollment by Study Wave [file jamanetwopen-e2510162-s002.pdf]

Supplementary Online Content

Keller P, Robertson T, Kao LS, et al. Incorporating behavioral science in medication adherence communication: a randomized clinical trial. *JAMA Netw Open*. 2025;8(5):e2510162. doi:10.1001/jamanetworkopen.2025.10162

eFigure. Participant Enrollment by Study Wave

This supplementary material has been provided by the authors to give readers additional information about their work.

© 2025 Keller P et al. *JAMA Network Open*.

eFigure. Participant enrollment by study wave

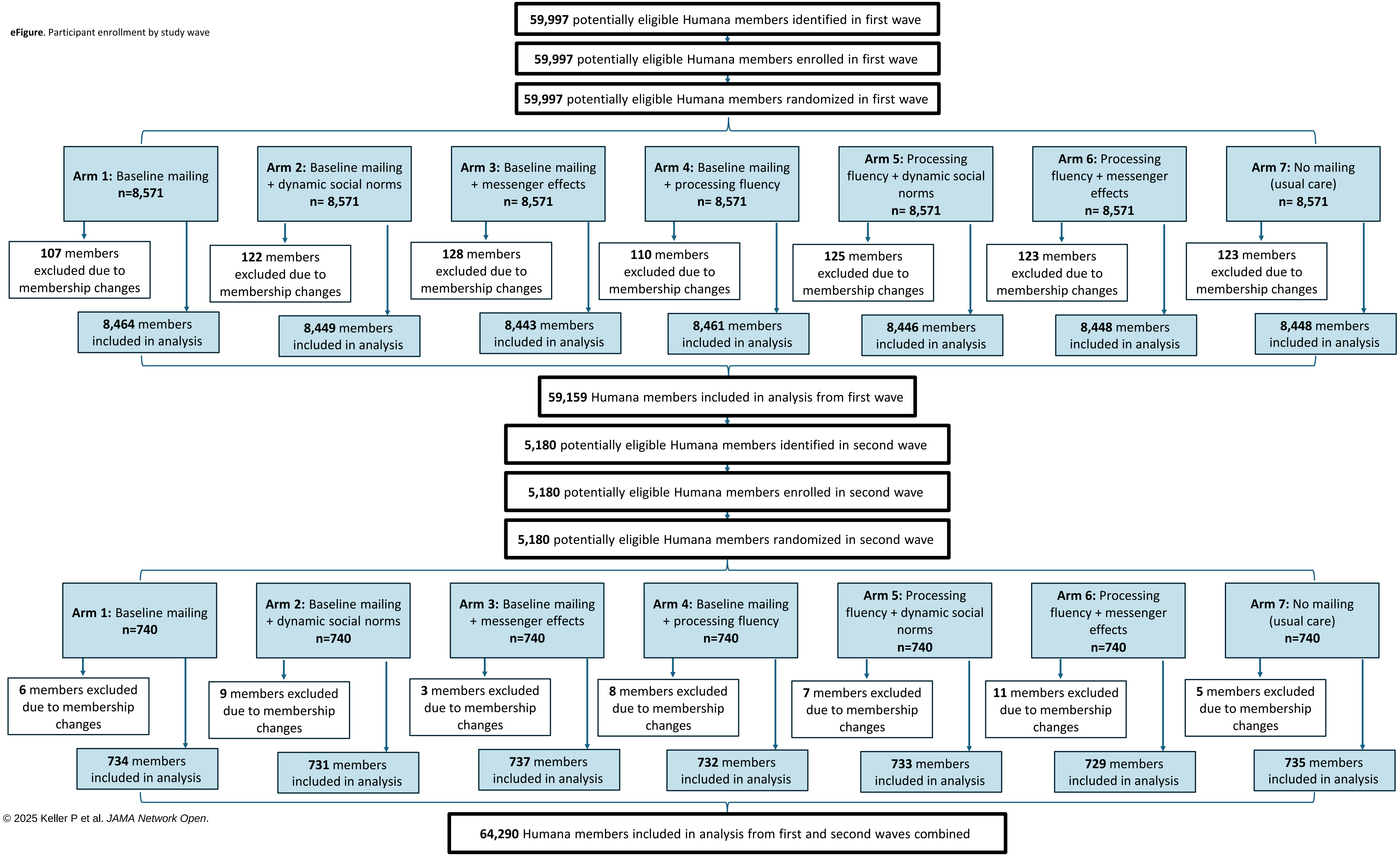

© 2025 Keller P et al. *JAMA Network Open*.
