# Supplementary material for: Incorporating Behavioral Science in Medication Adherence Communication: A Randomized Clinical Trial
Source: JAMA Netw Open. 2025 May 14;8(5):e2510162. doi: 10.1001/jamanetworkopen.2025.10162 (PMC12079288; doi:10.1001/jamanetworkopen.2025.10162)
Supplement: Supplement 3. — Data Sharing Statement [file jamanetwopen-e2510162-s003.pdf]

Data Sharing Statement

Keller. Incorporating Behavioral Science in Medication Adherence Communication. *JAMA Netw Open*. Published May 14, 2025. doi:10.1001/jamanetworkopen.2025.10162

Data
Additional Information: ClinicalTrials.gov: NCT06066541
Data available: No
